# Neuroinflammation in HIV-Related Neuropathic Pain

**DOI:** 10.3389/fphar.2021.653852

**Published:** 2021-04-20

**Authors:** Huan-Jun Lu, Yuan-Yuan Fu, Qian-Qi Wei, Zhi-Jun Zhang

**Affiliations:** ^1^ Institute of Pain Medicine and Special Environmental Medicine, Nantong University, Jiangsu, China; ^2^ Department of Human Anatomy, School of Medicine, Nantong University, Nantong, China; ^3^ Department of Infectious Diseases, General Hospital of Tibet Military Command, Xizang, China

**Keywords:** neuroinfalmmation, Chemokine, HIV, Human immunodeficiency virus, microglia, neuropathic pain (NP)

## Abstract

In the management of human immunodeficiency virus (HIV) infection around the world, chronic complications are becoming a new problem along with the prolonged life expectancy. Chronic pain is widespread in HIV infected patients and even affects those with a low viral load undergoing long-term treatment with antiviral drugs, negatively influencing the adherence to disease management and quality of life. A large proportion of chronic pain is neuropathic pain, which defined as chronic pain caused by nervous system lesions or diseases, presenting a series of nervous system symptoms including both positive and negative signs. Injury caused by HIV protein, central and peripheral sensitization, and side effects of antiretroviral therapy lead to neuroinflammation, which is regarded as a maladaptive mechanism originally serving to promote regeneration and healing, constituting the main mechanism of HIV-related neuropathic pain. Gp120, as HIV envelope protein, has been found to be the major toxin that induces neuropathic pain. Particularly, the microglia, releasing numerous pro-inflammatory substances (such as TNFα, IL-1β, and IL-6), not only sensitize the neurons but also are the center part of the crosstalk bridging the astrocytes and oligodendrocytes together forming the central sensitization during HIV infection, which is not discussed detailly in recent reviews. In the meantime, some NRTIs and PIs exacerbate the neuroinflammation response. In this review, we highlight the importance of clarifying the mechanism of HIV-related neuropathic pain, and discuss about the limitation of the related studies as future research directions.

## Introduction

Combination antiretroviral therapy (cART) has opened a new age in the treatment of HIV, in particular creating a new group of patients known as people living with HIV (PLWH). However, in addition to the immunodeficiency disease caused by HIV infection, various complications influence the mortality and treatment compliance among PLWH. According to the International Association for the Study of Pain (IASP), chronic pain is defined as pain that lasts or recurs for longer than 3 months, which is one of the consistently encountered symptoms in PLWH and is significantly associated with disability in daily activities, unemployment, and reduced quality of life (Ellis et al., 2010; Treede et al., 2019). Chronic neuropathic pain is chronic pain caused by nervous system lesions or diseases. Certain types of chronic pain are commonly identified as neuropathic pain, including spontaneous pain (continuous or episodic), hyperalgesia (exaggerated responses to normally painful), and mechanical allodynia (a painful response to a normally nonpainful stimulus) (Ji et al., 2003; Tsuda, 2018; Scholz et al., 2019). On clinical examination, the symptoms may present as positive signs (gain of somatosensory function), such as “pins and needles” sensation, painful ongoing sensation etc., or present as negative signs (loss of somatosensory function), such as distal sensory loss, reduced tendon reflexes, reduced temperature perception etc., but it is required that a history of nervous system injury or disease and a neuroanatomically plausible distribution of the pain to make the diagnosis (Tagliati et al., 1999; Gierthmuhlen and Baron, 2016; Scholz et al., 2019).

Neuroinflammation is a maladaptive mechanism responding to tissue injury in neuron system with the recruitment of immune cell and mediator releasing to promote regeneration and healing (Ellis and Bennett, 2013; Sommer et al., 2018). The combination and release of HIV-related glycoproteins (such as gp120) damage neurons, inducing neuroinflammation by a series of downstream alterations, increasing responses between neurons and glial cells and resulting in central sensitization and/or peripheral sensitization. These responses macroscopically present as chronic neuropathic pain in patients or rat models. Nucleoside analogue reverse transcriptase inhibitors (NRTIs) and protease inhibitors (PIs) that are used in the treatment of HIV also have neurotoxic effects, thereby exacerbating the neuropathic pain symptoms in PLWH as well. Recent reviews make a great introduction to the mechanism in chronic pain and clinical care for PLWH with chronic pain indicating that the inflammation response might play an important role in development of pain (Addis et al., 2020; Madden et al., 2020). In this review, we focus more on neuropathic pain and elucidate the mechanism of neuroinflammation in HIV-related neuropathic pain, for helping stimulate future researches about new targets to relief this torturous problem in PLWH.

### Epidemiology

The prevalence of chronic pain ranges from 25 to 90% (Newshan et al., 2002; Lee et al., 2009; Nair et al., 2009; Silverberg et al., 2009; Aouizerat et al., 2010; Cervia et al., 2010; Harding et al., 2010; Miaskowski et al., 2011; Merlin et al., 2012; Merlin et al., 2013; Parker et al., 2014; Lawson et al., 2015; Merlin et al., 2018), and some researches have highlighted the prevalence of neuropathic pain. 32% to more than a half of the participants reported neuropathic pain (Simpson et al., 2006; Nair et al., 2009; Robinson-Papp et al., 2009; Ellis et al., 2010). This variability can be partly attributed to different research methods, participants and definition of the origins of pain. Non-neuropathic pain, such as nociceptive pain, can be caused by tissue injury resulting from inflammation (e.g., autoimmune responses), infection (e.g., bacteria, other viruses, *tuberculosis*), or neoplasia (e.g., lymphoma or sarcoma) (Bruce et al., 2017). Considering the limitation of the questionnaire or self-report data, it is difficult to distinguish whether chronic pain is due to pathogen, neuropathy, or just cART induced. Additionally, patients with HIV-associated neuropathy are more than twice as likely to have other chronic pain disorders (Navis et al., 2018). Regardless of the types of pain, in fact more than 50% of PLWH have moderate to severe pain that affects daily life (Miaskowski et al., 2011; Merlin et al., 2012).

Certain demographic variables are associated with pain intensity, including female sex, non-Caucasian race, lower education level, and a history of drug use (Breitbart et al., 1996; Tsao et al., 2010; Miaskowski et al., 2011) (Table 1). Jiao et al. analyzed 638 patients and showed that 38% were affected by musculoskeletal pain, whereas 11% suffered from neuropathic pain including peripheral neuropathy (Jiao et al., 2016).

**TABLE 1:** Prevalence of pain, assessment method, and related factors in different chronic pain studies.

Articles	Prevalence of pain	Method of assessment	Influence factors	cART usage	Drug regimens
Merlin et al.; Merlin et al. (2018)	25% of 2,334 participants	Questionnaire (BCPQ Merlin et al.(2014) and PEG Krebs et al. (2009))	Long-term opioid therapy	All participants received HIV care services	No detailed description
Lawson et al. Lawson et al. (2015)	62.8% of 859	Questionnaire (Urwin Urwin et al. (1998))	Age, diagnosis time, PI regimen	76.5% on cART	*p*I/NNRTI/NRTI based
Miaskowski et al. Miaskowski et al. (2011)	270 of 296	Questionnaire (BPI Cleeland and Ryan, (1994) and PQAS Jensen et al. (2006))	Gender, education	74.4% on cART	No detailed description
Cervia et al. Cervia et al. (2010)	39% of 41	Self-reported pain scale data	cART	26% on cART	18 different kinds of regimens
Harding et al. Harding et al. (2010)	53.2% of 778	Questionnaire (MSAS) ( Chang et al. (2000)	Education	67.4% on cART currently	No detailed description
Aouizerat et al. Aouizerat et al. (2010)	55% of 317	Questionnaire (MSAS)	CD4^+^ T-cell count, race, sleep disturbance	71% on cART currently	No detailed description
Nair et al. Nair et al. (2009)	24.5% of 98 out-patients	Questionnaire (BPI)	cART	Partially usage	No detailed description
Lee et al. Lee et al. (2009)	55% of 317	Questionnaire (MSAS)	AIDS diagnosis, race, gender, cART	70% on cART	Include NRTI/PI based, or other combination therapy
Silverberg et al. Silverberg et al. (2009)	41.4% of 1,574 women	Self-report data	Race/ethnicity, age, depression and AIDS diagnosis	83.1% on cART of women	43.1% PI-containing of women
	43.1% of 955 men			90.8% on cART of men	50.1% PI-containing of men
Newshan et al. Newshan et al. (2002)	46% of 484	Clinic symptom checklist	None	No detailed description	No detailed description
Breitbart et al. Breitbart et al. (1996)	62% of 438	Questionnaire (BPI and MSAS)	HIV-related conditions, antiretroviral medications, age, and race	No detailed description	No detailed description

### The Mechanism Leading to HIV-Related Neuropathic Pain

Pathological pain in HIV patients is frequently associated with peripheral sensory neuropathy, which is a form of the so-called “dying-back” degeneration of sensory neurons (Hao, 2013). As the reasons for non-neuropathic chronic pain in diversified sites remain uncertain (e.g., musculoskeletal), the neuropathic pain related to HIV infection has been shown to be caused by neurotoxic effect of virus and the drugs applicated to treat HIV (Madden et al., 2020). Tough there is no existence of productive HIV infection in neurons, viral proteins do interact with neurons, glial cells, and immune cells both in central and peripheral nervous systems to inducing the development of neuroinflammation, macroscopic hyperalgesia and allodynia, a process that could be exacerbated by antiretroviral drugs.

### Viral Protein gp120 Is the Major Toxin Inducing the Neuropathic Pain

The HIV envelope glycoprotein gp120, together with gp41, undergoes receptor-driven conformational changes upon engaging the CD4 receptor and C-C chemokine receptor 5 (CCR5)/CXC chemokine receptor 4 (CXCR4) co-receptor binding for fusion of viral and host cell membranes (Acharya et al., 2015). As infection seldom occurs in neuronal cells, gp120 triggers hyperalgesia by directly activating the CXCR4 and/or CCR5 chemokine receptors in dorsal root ganglion (DRG) neurons (Oh et al., 2001). There are some reports that other HIV proteins such as Tat (transactivator of transcription) and Vpr (viral protein R) could also induce neuropathic pain, but a more convincing evidence suggests that gp120 is a major contributor to neuropathic pain (Acharjee et al., 2010; Chi et al., 2011; Yuan et al., 2014). Gp120 levels are about 10-fold higher in the spinal cord dorsal horn of pain-positive HIV patients than in that of pain-negative HIV patients. In contrast to gp120, Tat and Vpr, were not significantly elevated in the spinal cord dorsal horn of pain-positive HIV patients compared to pain-negative HIV patients (Yuan et al., 2014). Evidence supports that gp120 plays a direct role in the induction of allodynia. Symptomless HIV-1 infected patients who received an envelope subcomponent vaccine (MNrgp120) by intramuscular injection monthly report pain at the injection site in 1996 (Eron et al., 1996). Intrathecal injection of gp120 exacerbates pain in a mouse model, inducing robust thermal hyperalgesia and mechanical allodynia similar to the pathological phenotypes of pain-positive HIV-1 patients, followed by the expression of tumor necrosis factorα (TNFα) in the spinal cord with the activation of microglia and astrocytes (as discussed below) (Milligan et al., 2000; Milligan et al., 2001a; Herzberg and Sagen, 2001; Zheng W. et al., 2011; Yuan et al., 2014). Injection of TNFα induces neuropathic pain in humans (Eron et al., 1996; Wagner and Myers, 1996; Sorkin and Doom, 2000). Yi et al. elucidates the pathway of TNFα/TNF receptor 1 (TNFR1) –mitochondrial superoxide–pCREB triggers pC/EBPβ in HIV gp120-induced neuropathic pain state in rat model (Yi H. et al., 2018). HIV gp120 upregulates phosphorylated cAMP response element binding protein (pCREB) on the cytosine-cytosine-adenosine-adenosine-thymidine (CCAT)/enhancer binding protein ß (a member of the C/EBP family) gene promoter region *via* TNFR1 in neurons, which is a critical transcriptional regulator of HIV associated with cyclin-dependent kinase 9 (CDK9) and influences disease progression (Mameli et al., 2007; Yi Z. et al., 2018).

TNFα also plays a critical role in the wingless-type mammary tumor virus integration site family member 5 A (Wnt5a)/c-jun N-terminal kinase (JNK) signaling pathway induced by intrathecal injection of gp120. Wnt5a is upregulated in the spinal dorsal horn of HIV patients who develop pain and increased by intrathecal injection of HIV gp120 in rat neuropathic pain models. Inhibition of Wnt5a by specific antagonists blocks gp120-induced upregulation of interleukin-1β (IL-1β), IL-6, and TNFα in the spinal cord. The Wnt5a/Ca2+/Calmodulin-dependent Protein kinase II (CaMKII) pathway is critical for the gp120-induced expression of IL-1β, whereas the Wnt5a/JNK pathway is important for TNFα expression. The expression of IL-6 is co-regulated by both pathways (Li et al., 2013).

**FIGURE 1:**
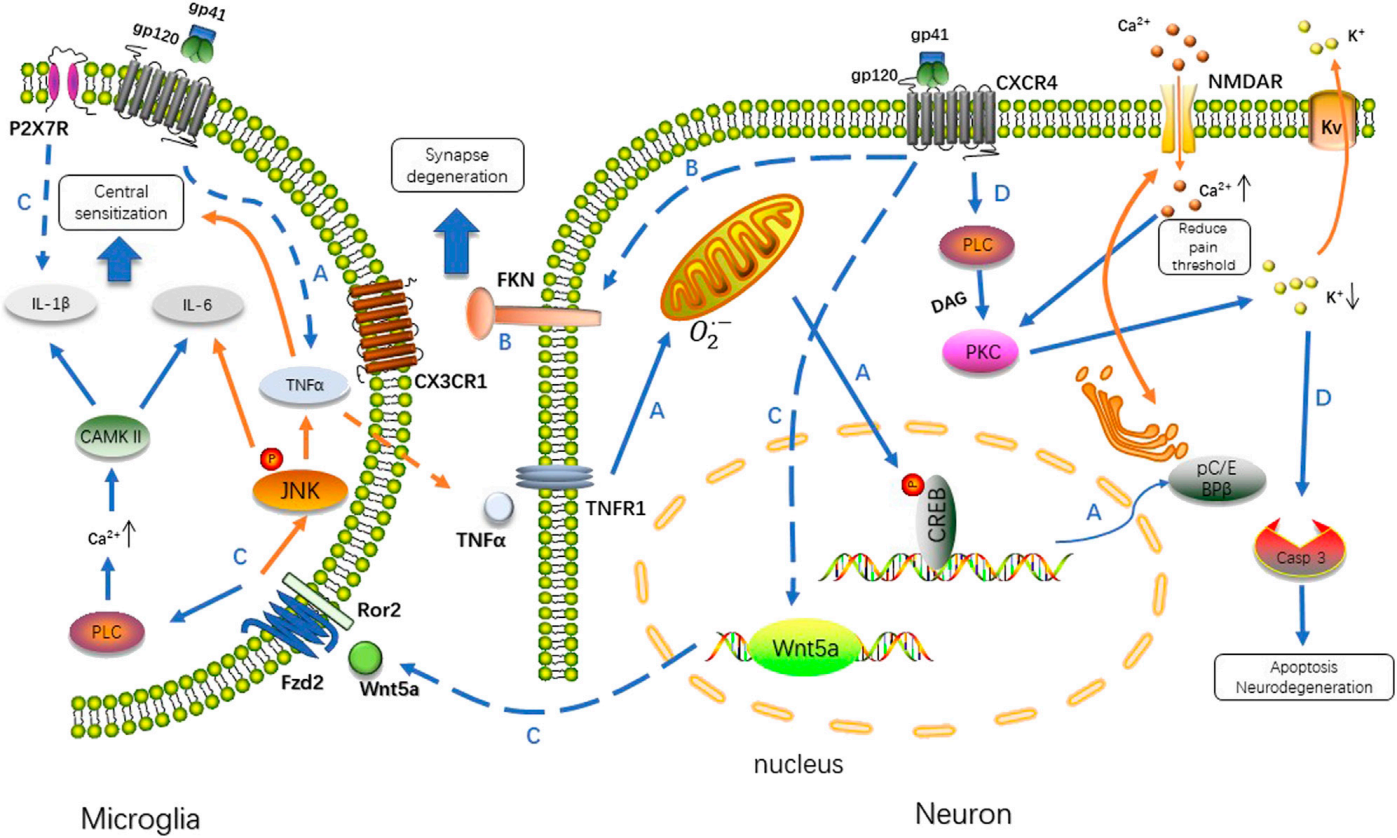
Gp120 binds to the CXCR4 receptor, and then triggers a series of downstream cellular signaling events involving TNFα/CREB/NMDAR, Wnt/β-catenin/FKN, Wnt5a/JNK/CAMKII and PLC/PKC/caspase 3 signal pathway. The interaction between neurons and microglia in response to infection induces neuropathic pain *via* different mechanisms. A. Stimulated by gp120, microglia up-regulate TNFα in a way of paracrine, TNFα/TNFR1 signal results in the accumulation of reactive oxygen species in mitochondria, which induced the phosphorylation of CREB triggering the pC/EBP (Zhang et al., 2004). C/EBP promotes recycling of NMDAR regulating the concentration of Ca2+ intracellularly (Wang et al., 2013; Hansen et al., 2018). B. FKN specifically expressed in neurons is upregulated by gp120, which activates microglia through CX3CR1. C. Transcription of Wnt5a is increased after gp120 application, stimulate the production of inflammatory cytokines in a paracrine fashion. Wnt5a/JNK is critical for TNFα expression, whereas Wnt5a/CaMKII is necessary for IL-1β and P2X7R is another factor predominant in driving IL-1β release. The expression of IL-6 is co-regulated by both pathways. D. Exposure of neurons to gp120 produces transient outward K+ currents in a PKC-dependent manner. The alteration of intracellular K+ homeostasis leads to neuronal apoptosis by activation of caspase-3. PLC, phospholipase C; DAG, diacylglycerol; PKC, protein kinase C; Casp 3, caspase 3; Fzd2, Frizzled family receptor 2; Ror2, receptor tyrosine kinase-like orphan receptor 2.

A gp120-Wnt5a-JNK-TNFα molecular axis is explored by the same group, indicating that Wnt5a potentiates the activity of spinal dorsal horn neurons *via* the JNK-TNFα pathway (Yuan et al., 2015). Similarly, gp120 combines with CXCR4 that increases cytomembrane outward K+ concentration by activating neuronal voltage-gated potassium (Kv) channels. Caspase-3 activation occurs downstream of the transient outward K^+^ currents, leading to neuronal injury (Chen et al., 2011). Gp120 triggers intracellular calcium alterations through a CXCR4-dependent mechanism to induce neuronal damage (Catani et al., 2000; Marchionni et al., 2012) (Figure 1).

### Central Sensitization and Peripheral Sensitization in HIV-Related Neuropathic Pain

Triggered by nerve damage, including infection, inflammation or drug toxicity, neuropathic pain is involved in both central sensitization, increased responsiveness of nociceptive neurons in the central nervous system (CNS) to their normal or subthreshold afferent input, and peripheral sensitization, increased responsiveness and reduced threshold of nociceptors to stimulation of their receptive fields in the peripheral nervous system (PNS), inducing the development and maintenance of hyperalgesia and allodynia (Loeser and Treede, 2008; Gao and Ji, 2010). Driven by the release of cytokines, chemokines and neurotransmitters, the activation of non-neuronal cells such as immune cells and glial cells in both CNS (e.g., microglia and astrocytes) and PNS (e.g., macrophages, Schwann cells and satellite cells) plays an important role in neuropathic pain progressing (Huang et al., 2013; Silva et al., 2017; Lee et al., 2018; Liu et al., 2019). Macrophages and microglia express high levels of CD4 and CCR5 and are therefore a prime target for HIV infection, forming a reservoir for reactivation of virus replication. Noteworthy, Intact HIV and its viral protein gp120 could cross the blood-brain-barrier (BBB) *via* transcytosis or paracellularly, infecting both microglia and astrocytes while in this process, gp120 might be a key in determining whether free virus can cross the BBB (Banks et al., 2001; Hong and Banks, 2015). Noxious factors released from immune-activated, HIV-infected, or gp120-stimulated macrophages and microglia may damage peripheral or central pain transmission neurons directly or through an associated cascade in proinflammation pathways, both of which induce hypersensitivity and allodynia related to neuropathic pain. Here we will mainly discuss the mechanism of central and peripheral sensitization induced by HIV infection leading to neuropathic pain.

### Central Sensitization

There are accumulating evidences supporting the critical role of microglia in central sensitization. Microglia are CNS-resident macrophages described as “neuroimmune sentinels” that orchestrate the immune response to invading pathogens, toxins, and cellular debris. Microglial activation exhibits morphological changes from “resting microglia” state and can assume neurotoxic or neuroprotective priming states that determine their responses to danger (Hickman et al., 2013; Qin et al., 2019). Evidences support that microglia act as a key mechanism in the development and maintenance of neuropathic pain, not only in HIV-related neurological injury, but in spinal or sciatic nerve injury and trigeminal neuropathic pain (Ji et al., 2013; Inoue and Tsuda, 2018; Xin, 2019). In response to HIV infection, the rapid activation of microglia may also release neurotoxic factors, including proinflammatory cytokines such as TNFα, IL-1β, IL-6 (Milligan et al., 2001a; Schoeniger-Skinner et al., 2007; Liu et al., 2016), chemokines such as monocyte chemoattractant protein-1 (MCP-1), fractalkine (FKN) (Zhao et al., 2017; Ru et al., 2019), excitatory amino acids (Porcheray et al., 2006; Tian et al., 2012), nerve growth factors such as brain-derived neurotropic factor (BDNF) (Wang et al., 2017), and reactive oxygen species (Patrizio and Levi, 1994; Holguin et al., 2004). These factors modulate neuroinflammation, which plays an important role in neuropathic pain maintenance compared with systemic inflammation (Ji et al., 2016). In an HIV-1SF162 infected primary human microglial model, El-Hage et al. showed the secretion of IL-8, IL-6, MCP-1, TNFα, and CCL5 is increased in cell culture supernatants (El-Hage et al., 2015). P2X7, a subtype of ionotropic P2X receptors, is stimulated by extracellular ATP signals and involved in neuropathic pain. After nerve injury, P2X7 mRNA is predominantly expressed in spinal microglia (Kobayashi et al., 2011). The release/production of IL-1β by microglia may be related to overexpression of the purinergic P2X7 receptor, which is associated with microglial activation and proliferation (Monif et al., 2016). These cytokines collectively shape the inflammatory response to HIV infection, form a persistent pathological change, and then promote the occurrence of central sensitization in neuropathic pain underlying PLWH.

PLWH suffering from neuropathic pain may have a decrease in the epidermal nerve fiber density as well as neurodegenerative changes (Phillips et al., 2014; Yuan et al., 2014; Mawuntu et al., 2018). Synapse loss often occurs in pain-positive HIV patients. Intrathecal injection of gp120 in the spinal dorsal horn causes a significant decrease of synapse markers such as synapsin I (Syn I), postsynaptic density-95 (PSD-95), and NMDA receptor subunit 1 (NR1). These results suggest that synaptic degeneration is closely connected with HIV-related neuropathic pain (Yuan et al., 2014). Microglia modulate the formation of neuronal synapses and regulate neuronal activity *via* phagocytosis (Paolicelli et al., 2011; Hong et al., 2016). FKN/CXC3R1 (an FKN receptor predominantly expressed in microglia) signaling-mediated neuron-microglia cross-talking plays a critical role in HIV infection-induced synaptic degeneration (Ru et al., 2019). In gp120-treated primary cortical cultures, the expression of the FKN protein in neurons was gradually upregulated over time. Knockout of CX3CR1 restored gp120-induced alteration in synaptic proteins such as PSD-95 and Syn I. This process was triggered by the Wnt/β-catenin pathway. Gp120 induces FKN expression and synapse loss *via* NMDA receptor activation which is often thought to participate in the procedure of presynaptic inhibitory (Lotankar et al., 2017). The NMDAR antagonist APV, Wnt/β-catenin signaling suppressor DKK1, or knockout of CX3CR1 alleviates gp120-induced mechanical allodynia in mice, suggesting that the Wnt/β-catenin-FKN/CX3CR1 pathway mediates synaptic degeneration associated with HIV neuropathic pain (Ru et al., 2019). The FKN/CX3CR1 pathway is also discussed in recent review of Huang et al. toughly (Huang et al., 2020).

Astrocytes are numerous CNS glial cells, contributing actively to the formation and maintenance of BBB, and form an intricate crosstalk with neurons and immune cells by neurotransmitters, inflammatory cytokines or chemokines. (Rothhammer and Quintana, 2015; Ben and Rowitch, 2017; Linnerbauer et al., 2020). Similarly, astrocyte infected by HIV is generally non-productive but of which reaction is more persistent and occurs in more painful conditions in spinal cord compared with the rapid and dramatic microglial reaction (Gorry et al., 2003; Ji et al., 2018). Astrocytes are activated by gp120 and Tat to produce inflammatory cytokines such as TNFα, IL-1 and IL-6, the chemokine CCL5, and constitutive nitric oxide synthase (cNOS), which is a mediator for the production of pain facilitation (Togashi et al., 1997; Milligan et al., 2001b; Shah et al., 2011a; Shah et al., 2011b; Zheng X. et al., 2011; Sanchis et al., 2020). These chemicals facilitate neuropathic pain *via* influencing the microenvironment that is essential for neuronal and glial function. The process could be bilateral. For instance, increased TNFα induced MCP-1 expression in spinal cord astrocytes in a JNK-dependent manner, and its production could promote astrocyte glutamate release, both indicating the development of mechanical allodynia (Bezzi et al., 2001; Gao et al., 2010). Reciprocally, astrocytes control the transcriptional programs of microglia and CNS-infiltrating monocytes in a non-cell autonomous manner by regulating granulocyte-macrophage colony-stimulating factor (GM-CSF) (Mayo et al., 2014).

**FIGURE 2:**
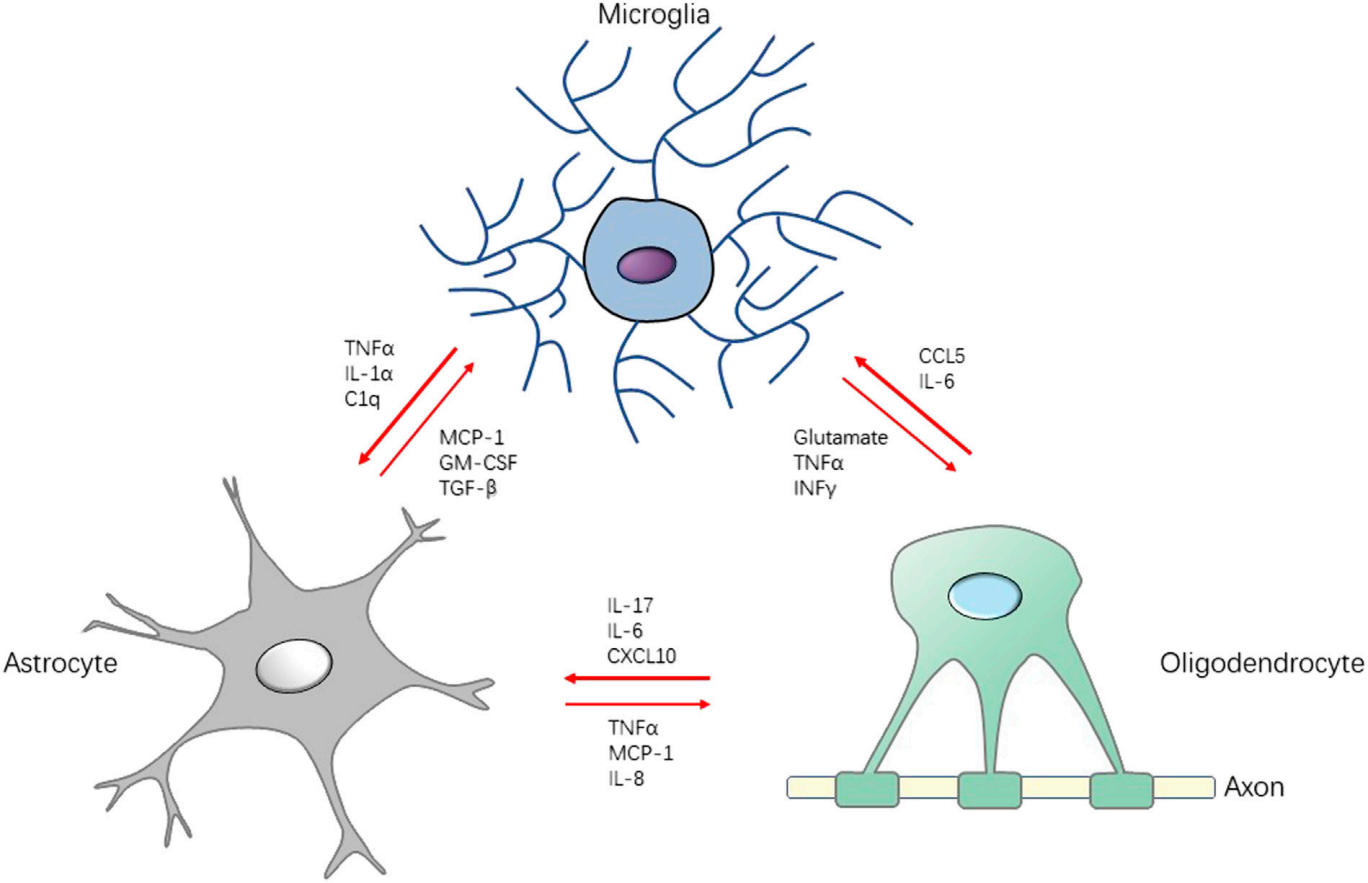
Schematic diagram for the interaction between microglia, astrocytes and oligodendrocytes in neuroinflammation. Stimulated by pathogen or injury, the production of cytokines, chemokines, and growth factors by microglia may induce activation on astrocytes and oligodendrocyte, which accelerates the neuroinflammation in CNS, leading to central sensitization. Conversely, there are some factors (e.g., TGF-β, IL-10) that attenuate the development of neuroinflammation, serving the function of neuroprotection. TGF-β: transforming growth factor-β; INFγ: interferon γ.

Oligodendrocytes are generally considered as myelinating cells of providing continuous metabolic and trophic support to neurons, believed to be simply victims of the inflammatory reaction (Nave and Trapp, 2008; Zeis and Schaeren-Wiemers, 2008). However, a growing body of evidence suggests that oligodendrocytes take a more active part in immunomodulatory process. It has been reported that oligodendrocytes express cytokines (IL-17A, IL-18, IL-6) (Cannella and Raine, 2004; Tzartos et al., 2008; Ramesh et al., 2012), chemokines (MCP-1, CCL5) (Balabanov et al., 2007; Moyon et al., 2015), complement (C2, C3) (Hosokawa et al., 2003) to participate the communication network between microglia and astrocytes. A study indicates that activation of microglia is followed by astrocyte activation which contributes to oligodendrocyte cell death. This tri-glial dysregulation is dependent on microglia (Gibson et al., 2019). In pain-positive HIV patients, cell markers of the oligodendrocyte lineage, including NG2, PDGFRα, and Olig2, are significantly increased (Shi et al., 2016). These evidences reveal that the activation of the network among microglia, astrocytes and oligodendrocytes is the important neuropathic pain mechanism (Figure 2).

### Peripheral Sensitization

Peripheral nerves are the origin of almost all forms of neuropathic pain (Ramesh et al., 2013). In response to viral infection or peripheral nerve injury, resident macrophages and peripheral glia cells are early activated prior to central ones, exhibiting diverse mechanisms that lead to peripheral sensory neurons sensitization (Ji et al., 2016). As discussed by Addis et al., macrophages traffic to DRG and shape the peripheral neuron inflammatory milieu after HIV infection. By recruiting T helper type 1 (Th1) cells or Th2 cells, macrophages display pro-inflammatory (M1) or pro-resolution (M2) phenotype (Addis et al., 2020). M1 macrophages are characterized by high expression of pro-inflammatory cytokines such as TNFα, IL-6, IL-1β, which induce local neuro-inflammation, leading to the establishment of neuropathic state. Reversely, M2 macrophages produce anti-inflammatory cytokine IL-10. The enhancement of IL-10 expression or inhibition of M1 macrophages could attenuate or abolish the neuropathic pain state (Kiguchi et al., 2017; Fonseca et al., 2019; Iwasa et al., 2019). Schwann cells are important glial cells in the peripheral nervous system, which act as insulators of axons. Not only Schwann cells provide metabolic and trophic support for peripheral nerve, but also modulate response to nerve injury (Kim et al., 2018; Sasaki et al., 2018). Though previous works about gp120 interacts with Schwann cells inducing subsequently release of chemokines, resulting in apoptosis of DRG *via* CCR5 (Keswani et al., 2003; Melli et al., 2006). Recent studies have aroused interests in the function of Schwann cells in HIV-related neuropathic pain from a new perspective. After T-cell line tropic X4 strain gp120 application (infects T-cell lines *via* CXCR4 but not macrophages), circulating macrophage infiltration into the peripheral nerves induces neuropathic pain. This phenomenon could be caused by the expression of CXCL1, a chemoattractant of macrophages and neutrophils, which is derived from gp120-treated cultured Schwann cells but not neurons (Ntogwa et al., 2020). Furthermore, gp120 increases lysosomal exocytosis and enhances release of ATP in primary human Schwann cells by the activation of P2X4 cationic channels. As a consequence, gp120-induced lysosomal exocytosis of ATP in Schwann cells elevated intracellular Ca2+ in DRG neurons, leading to HIV-related neuropathic pain (Datta et al., 2019). Satellite glial cells (SGCs) surround the bodies of DRG neurons and form close shells *via* gap junctions (Hanani, 2005; Costa and Moreira, 2015). SGCs contribute to neuropathic pain in a way that produce cytokines like IL-1β, matrix metalloprotease-2 (MMP-2) (Kawasaki et al., 2008) and modulate the expression of ionotropic P2X receptors and metabotropic P2Y receptors like P2X4, P2X7, P2Y12 (Chen et al., 2008; Yi H. et al., 2018; Zhao et al., 2019).

### Antiretroviral Drugs Lead to Neuropathic Pain

The development and increased availability of HAART have dramatically reduced HIV-related morbidity and mortality, restricting HIV as a chronic, inflammatory disease. The prolonged use of cART is associated with the development of neurological disorders despite decreased HIV loads (Brew et al., 2009; Madden et al., 2020). One common neurological disorder induced by side-effect of cART is neuropathic pain.

Certain NRTIs including stavudine (d4T), zidovudine (AZT), didanosine (ddI), and zalcitabine (ddC) are neurotoxic and pivotal for inducing neuropathic pain (Manji, 2000; Yuan et al., 2018). D4T and ddC are notorious among these NRTI drugs and their toxicity has been studied both *in vitro* and *in vivo*. D4T is widely used as the first-line regimen in low-income countries with limited resources (Loubiere et al., 2010; Asgedom et al., 2020). D4T-based ART increases the risk of peripheral neuropathy, hyperlactatemia, and other diseases (Menezes et al., 2011). Intraperitoneal injection of d4T or ddC in rat induced mechanical allodynia and cold allodynia (Wallace et al., 2007; Zheng W. et al., 2011; Sanna et al., 2016). Recent study reveals that the expression of 135 genes in mice given injection of ddC has significant changes mainly enriched in regulation of transcription, multicellular organism development, and cell differentiation *via* transcriptome sequencing (Wu et al., 2021). Exposure to ddC upregulates the pro-nociceptive chemokine MCP-1, stromal cell-derived factor-1 (SDF-1), and TNFα in DRG (Bhangoo et al., 2007; Wallace et al., 2007; Zheng X. et al., 2011). AZT/lamivudine/d4T administration in mice increased TNFα, IL-1β and IL-6 in various CNS regions *via* a Wnt5a-dependent mechanism (Wu et al., 2017). A direct evidence reported by Yuan et al. suggests that ddC induces neuroinflammation in the spinal cord, with the up-regulation of TNFα and IL-1β. It activates astrocytes and microglia, resulting in allodynia in mice model, which is regulated by spinal Wnt5a (Yuan et al., 2018).

PIs are another cause of neuropathic pain. In HIV seropositive patients, indinavir, saquinavir, and ritonavir are associated with neuropathic pain, and indinavir shows selective cytotoxicity to macrophages in DRG, inducing neuronal atrophy and neurite retraction (Pettersen et al., 2006). Adult rats treated with indinavir develop hind paw mechanical hypersensitivity independent of HIV infection. Treatment with PI activates microglia in the lumbar spinal dorsal horn by inducing the phosphorylation of p38 in microglia, which mimics the clinical features of PI-treated HIV patients (Huang et al., 2017).

### Future Perspective

The prevalence of neuropathic pain varies widely among HIV populations. Take those influence factors described in the chronic pain research into consideration, including gender, race, PI or neurotoxic NRTI drugs usage and cART regimens, high stand of screening criteria and methods should be established to obtain a more accurate epidemiology data. As most works are done by intrathecal injection of gp120 in rat model to simulate the neuropathic pain state in PLWH, which is an exogenous stimulus originated from HIV envelope proteins, whether this method could actually present the neuroinflammation occurs in pain-positive patients is still remain discussion. Actually, immune cells express inflammation modulatory factors (e.g., IL-10 and IL-27) during different phases of HIV infection (Soare et al., 2019). IL-27 receptor, expressed by macrophage, microglia, and astrocytes of the sensory ganglia and spinal cord, its activation has the function of counteracting neuropathic pain (Fonseca et al., 2019). Therefore, both pro-inflammation and anti-inflammation are processing simultaneously between immune cells. It is interesting to investigate the crosstalk between microglia, astrocytes and oligodendrocytes during HIV infection resulting neuropathic pain, which microglia could play a center role of the neuroinflammation. The cellular tropism of HIV strains applicated in the experiment is neglected in the early work. But the study of Ntogwa et al. raises an important question that the signal pathway activated by X4 gp120 between Schwann cells and macrophages is not involved in the development of R5 gp120-induced neuropathic pain. The role of macrophage in X4 gp120-induced neuroinflammation is not yet fully understood (Ntogwa et al., 2020). It will be critical to do more researches about the neuroinflammation during HIV infection, and identify important targets to design related medication for blocking the progression of neuropathic pain.

## Abbreviations

BBB, blood-brain-barrier; BDNF, brain-derived neurotropic factor; CCAT, cytosine-cytosine-adenosine-adenosine-thymidine; CaMKII, Calmodulin-dependent Protein kinase II; cART, combination antiretroviral therapy; CDK9, Cyclin-dependent kinase 9; CCR5, C-C chemokine receptor 5; cNOS, constitutive nitric oxide synthase; CNS, central nervous system; CXCR4, CXC chemokine receptor 4; DRG, dorsal root ganglion; FKN, fractalkine; GM-CSF, granulocyte-macrophage colony-stimulating factor; HIV, human immunodeficiency virus; IL-1β, interleukin-1β; JNK, c-jun N-terminal kinase; MCP-1, monocyte chemoattractant protein-1; NR1, NMDA receptor subunit 1; NRTIs, Nucleoside analogue reverse transcriptase inhibitors; pCREB, cAMP response element binding protein; PIs, protease inhibitors; PLWH, people living with HIV; PNS, peripheral nervous system; PSD-95, postsynaptic density-95; SDF-1, stromal cell-derived factor-1; Syn I, synapsin I; Tat, transactivator of transcription; TNFR1, TNF receptor 1; Vpr, viral protein R; Wnt5a, wingless-type mammary tumor virus integration site family member 5A.
